# Factors Related to the Well-Being of Older Chinese Living in Institutional Care Facilities: A Systematic Review

**DOI:** 10.1093/geroni/igaa057.589

**Published:** 2020-12-16

**Authors:** Kun Wang

**Affiliations:** University of Alabama, Tuscaloosa, Alabama, United States

## Abstract

Due to the fast pace of population aging and the “4-2-1” family structure, institutional care was proposed as “support” for the elderly care service system in China. The purpose of this systematic review paper was to identify factors that are associated with the well-being of older residents living at institutional care facilities in China. Studies were included if participants (1)aged 60 years or older, (2) were living at an institutional care facility in mainland China. Studies were excluded if participants (1) were Chinese Immigrants, or residents in Hong Kong, Macao, and Taiwan, (2) were cognitively impaired, or (3) at the end of their lives and need palliative care in institutional facilities. A total of 12 articles were selected in this review study based on PRISMA guidelines: 10 quantitative studies and 2 qualitative studies. Anderson healthcare utilization model was used in this study to categorize related factors into three dynamics: predisposing factors, enabling factors, and need factors. Among predisposing factors, older, more educated, widowed adults with higher income were more likely to have higher levels of well-being in institutional care facilities. Social supports, such as family visit, activity engagement and peer support, were very important enabling factors. The actual need, such as ADL, health status and depression, was another important dimension for the well-being of older Chinese living in institutional care facilities. Aiming at increasing older residents’ well-being, the present study suggested more tailored interventions should be designed and implemented to enhance their social support, activity engagement and peer support.

